# Polysaccharides from the Medicinal Mushroom *Cordyceps taii* Show Antioxidant and Immunoenhancing Activities in a *D*-Galactose-Induced Aging Mouse Model

**DOI:** 10.1155/2012/273435

**Published:** 2012-03-29

**Authors:** Jian-Hui Xiao, Dai-Min Xiao, Dai-Xiong Chen, Yu Xiao, Zong-Qi Liang, Jian-Jiang Zhong

**Affiliations:** ^1^Division of Applied Mycology and Biochemical Pharmacy, Key Laboratory of Cell Engineering of Guizhou Province, Affiliated Hospital of Zunyi Medical College, Zunyi 563000, China; ^2^State Key Laboratory of Microbial Metabolism, and School of Life Science & Biotechnology, Shanghai Jiao Tong University, Shanghai 200240, China

## Abstract

*Cordyceps taii*, an edible medicinal mushroom native to south China, is recognized as an unparalleled resource of healthy foods and drug discovery. In the present study, the antioxidant pharmacological properties of *C. taii* were systematically investigated. *In vitro* assays revealed the scavenging activities of the aqueous extract and polysaccharides of *C. taii* against various free radicals, that is, 1,1-diphenyl-2-picrylhydrazyl radical, hydroxyl radical, and superoxide anion radical. The EC_50_ values for superoxide anion-free radical ranged from 2.04 mg/mL to 2.49 mg/mL, which was at least 2.6-fold stronger than that of antioxidant thiourea. The polysaccharides also significantly enhanced the antioxidant enzyme activities (superoxide dismutase, catalase, and glutathione peroxidase) and markedly decreased the malondialdehyde production of lipid peroxidation in a *D*-galactose-induced aging mouse model. Interestingly, the immune function of the administration group was significantly boosted compared with the *D*-galactose-induced aging model group. Therefore, the *C. taii* polysaccharides possessed potent antioxidant activity closely associated with immune function enhancement and free radical scavenging. These findings suggest that the polysaccharides are a promising source of natural antioxidants and antiaging drugs. Consequently, a preliminary chemical investigation was performed using gas chromatography-mass spectroscopy and revealed that the polysaccharides studied were mainly composed of glucose, mannose, and galactose. Fourier-transform infrared spectra also showed characteristic polysaccharide absorption bands.

## 1. Introduction

Aging, an extremely complex biological phenomenon, is commonly defined as the progressive accumulation in an organism of diverse, deleterious changes with time that increase the chance of disease and death [1]. However, why and how does aging occur? There is controversial for the aging process of organisms, and more than 300 theories have been proposed to explain the aging process [2]. Among these theories, the free radical theory of aging is recognized as one of the most plausible and promising explanations for the process of aging [3]. The theory, conceived in 1956 by Denham Harman who suggested that the free radicals generated during aerobic respiration, would cause cumulative oxidative damage, resulting in aging and death [4]. Free radicals are molecules or molecular fragments containing one or more unpaired electrons in atomic or molecular orbitals and generally occur through different pathways due to exogenous chemical and endogenous metabolic processes in living systems. Generally, only overproduction free radicals can cause potential biological damage to nucleic acids, lipids, and proteins in the body. Whereas excessive amount of free radicals is the result of an imbalance between free radical production and antioxidant defense in the body, which is called as “oxidative stress” [5, 6]. Oxidative stress can lead to cell and tissue damage and to the dysregulation of redox-sensitive signaling pathways, and there are growing evidences that oxidative stress increases with aging [7]. Also, scientific studies have found that aging is closely associated with a decline in immune function known as immunosenescence [8, 9]. Moreover, the immune system has been proposed as a marker of biological age and life span since a suboptimal immune function may significantly contribute to morbidity and mortality in the elderly [10]. Accordingly, the decrease in the function of immune system is possibly associated with the emergence of excessive free radicals during the aging process. For example, a strong parallelism between oxidizing species generation and cellular reducing power response in human granulocytes was reported by Chaves et al. [11], in which the aging groups showed an increase in the free radicals generation and a significant decrease in the cellular reducing power. Based on the free radical theory, however, further research is still needed to elucidate the correlation between the aging-associated decline in immunity and free radical formation. For aging-associated free radicals, antioxidant plays an important role to prevent from oxidative stress caused by free radicals, which can safely interact with free radicals and terminate the chain reaction before vital biological macromolecules are damaged. Humans possess a good antioxidant defense network comprising the well-known endogenous enzymes superoxide dismutase (SOD), catalase (CAT), and glutathione peroxidase (GSH-Px), as well as nonenzymatic antioxidants such as ascorbic acid, tocopherol, reduced glutathione (GSH), and melatonin, which participate in maintaining the proper balance of free radicals and antioxidants in the health body. Under abnormal physiological and/or pathological conditions, however, the antioxidant defense system is insufficient for completely preventing oxidative damage caused by excessive free radicals, which results in oxidative stress. Fortunately, there is a convincing scientific evidence that some exogenous chemicals may be used as antioxidants to attenuate oxidative damage. For example, antioxidants could exert important favorable effects improving the immune function, as well as decreasing the oxidative stress [8, 10, 12], Consequently, great interest has arisen in the possibility that antioxidants, particularly naturally occurring antioxidants from edible materials, may reduce the risk of aging and aging-related degenerative diseases [13, 14]. Numerous antioxidants, such as phenolic compounds (e.g., polyphenols, flavonoids, and tannins), carotenoids, organic acid, vitamins, and polysaccharide discovered from various natural sources have already been utilized in foods, medicines, and cosmetics [14–17].

Edible and medicinal mushrooms are rich in dietary fiber, nutrients, and other compounds known to be physiologically beneficial to humans. These mushrooms are important sources of natural antioxidants and have become a very active domain of research in recent years [18–21]. The antioxidative potentials of mushrooms are attributed to such chemical constituents as phenolic compounds, carotenoids, organic acids, polyketides, and peptides [20, 21]. *Cordyceps* is a well known and valued traditional Chinese medicinal mushroom. It has been widely used for centuries in China as a tonic for promoting vitality and longevity, as well as a herbal medicine for treating various intractable diseases [22]. One of its known pharmacological actions is antioxidant activity. Previous studies have indicated that the aqueous extracts of natural and cultured *Cordyceps* species (e.g., *Cordyceps sinensis* and *Cordyceps militaris*) show antioxidant activities *in vitro*, including free-radical scavenging, lipid peroxidation inhibition, and metal ion chelation [23–25]. Interestingly, in partially purified polysaccharide fractions from cultured *C. sinensis*, antioxidant activities increase from 10-fold to 30-fold [23]. This finding suggests that polysachacrides in *Cordyceps* may be major antioxidative substances, as recent investigations have demonstrated [14, 17, 23]. However, to our knowledge, the antioxidative potential of the *Cordyceps* species has not yet been evaluated *in vivo*. Therefore, experiments for determining the possible use of *Cordyceps *species as natural antioxidant sources in an *in vivo* model are urgently required.

*Cordyceps taii*, a new species native to south China, has been discovered and identified by our group in the 1990s [26]. This species has been used as a folk medicinal mushroom for a long time. The fermentation conditions of *C. taii*, as well as its corresponding chemical composition and chemical markers, have been identified [22, 26, 27]. Pharmacological experiments further reveal that *C. taii* has broad-spectrum antimicrobial effects, including potent antibacterial and antifungal ones. Fractions extracted with organic solvents such as chloroform, ethyl acetate, and acetone are rich in active ingredients [22]. Aqueous polysaccharides from *C. taii* also display different bioactivities such as antitumor, and antimutagenic [26]. The optimal conditions for the large-scale preparation of *C. taii* polysaccharides have also been investigated in recent time [28]. Despite these extensive studies, the antioxidant effects of the aqueous polysaccharides of *C. taii* have not yet been reported. These effects need to be well understood to reveal the true potential of *C. taii* against free radicals. In the present study, the antioxidative potential and immunoregulatory function of aqueous polysaccharides from *C. taii* were systematically developed in *D*-galactose-induced aging mouse model for the first time.

## 2. Materials and Methods

### 2.1. Chemicals and Reagents

1,1-Diphenyl-2-picrylhydrazyl (DPPH), concanavalin A (ConA), lipopolysaccharide (LPS), butylated hydroxytoluene (BHT), and butyl hydroxyanisole (BHA) were purchased from Sigma Inc. (St. Louis, MO, USA). RPMI 1640 with *L*-glutamine was from GIBICO BRL Life Technologies, Inc. (Grant Island, NY, USA). EZ-sep Mouse 1X Lymphocyte Separation medium was from Dakewe Inc. (Shenzhen, China). The cell proliferation 3-(4,5-dimethylthiazol-2-yl)-5-(3-carboxymethoxyphenyl)-2-(4-sulfophenyl)-2*H*-tetrazolium (MTS) assay kit was from Genmed Scientifics Inc. (Genmed, Shanghai, China). Ethylenediamine tetraacetic acid (EDTA) and dimethyl sulfoxide were from Invitrogen Inc. (Carlsbad, CA, USA). Trichloroacetic acid, ferrozine, potassium ferricyanide, ferrous chloride and ascorbic acid were from Advanced Technology & Industrial Co., Ltd. (Hongkong, China). Ortho-phenanthroline (1,10-phenanthroline) was from Damao Chemical Reagents Factory (Tianjin, China). Thiourea (TU) was from Kexing Biotechnology Co., Ltd. (Shanghai, China). Diethylaminoethyl- (DEAE-) cellulose-52 and Sephadex G-100 were from GE Healthcare (Pharmacia, Sweden). The detection kits for superoxide anion radical scavenging activity, antioxidative enzymes (SOD, CAT, GSH-Px), and malondialdehyde (MDA) were from the Nanjing Jiancheng Bioengineering Institute (Nanjing, China). All chemicals used were of analytical grade.

### 2.2. Medicinal Fungus, Media, and Sample Preparation

The voucher specimens of *C. taii* (strain GYYA 0601) were deposited at the Laboratory of Applied Mycology and Biochemical Pharmacy, Zunyi Medical College, Guizhou Province, China. *C. taii* was maintained on a slant seed medium (2.5% w/v sucrose, 0.3% soybean, 0.5% yeast extract, 0.2% peptone, 0.3% wheat bran, 0.1% KH_2_PO_4_, 0.05% MgSO_4_·7H_2_O, 1.8% agar, and 1 L of distilled water; initial pH = 6.0). The stock culture was incubated at 28°C for 15 d and then stored at 4°C in a refrigerator before use. As previously described [28], a liquid seed culture was performed on a rotary shaker incubator. The fermentation medium (3% sucrose, 0.5% soybean, 0.2% soybean extract, 0.5% yeast extract, 0.1% KH_2_PO_4_, 0.05% MgSO_4_, and 1 L of distilled water; initial pH = 6.0) was inoculated with 3% to 5% (v/v) of the liquid seed culture and then cultivated at 28°C in a 300 mL flask containing 150 mL of the medium on a rotary shaker incubator at 150 rpm for 6 d to 7 d. The mycelia of *C. taii* were harvested by vacuum filtration and thrice washed with distilled water. Subsequently, the mycelia were lyophilized and grinded (60 mesh to 100 mesh) for later experiments.

### 2.3. Isolation and Fractionation of Aqueous Polysaccharides

Dry power of cultured *Cordyceps* mycelia (1 kg) was repeatedly defatted with petroleum ether at 60°C. The residue was obtained by centrifugation (5000 ×g for 15 min), dried at room temperature, and then repeatedly extracted by chloroform as well as acetic ether (in that order). Subsequently, the residue was extracted at 95°C with distilled water for 2 h. After vacuum filtration, the residue was re-extracted five times under the same condition. The filtrates were combined and condensed to one fifth of their total volume using a rotary evaporator under reduced pressure at 50°C to 55°C. The filtrates were then precipitated at 4°C for 12 h with ethanol to an 85% final concentration. The precipitate was thrice washed with 70% ethanol and lyophilized in a vacuum, yielding the aqueous extract of* C. taii* (AECT), which was stored at 4°C before use. The AECT was redissolved with distilled water, depigmented by the H_2_O_2_ method, deproteinized by the Sevag method, and dialyzed against distilled water for 3 d. The nondialyzable phase was reconcentrated and then fractionally precipitated using an ethanol solution to an 85% final concentration. The resulting precipitate was collected by centrifugation, thrice washed with acetone, and dried to a constant weight. Finally, the aqueous polysaccharide fraction of *C. taii* (APCT) was obtained and was stored at 4°C before use. By the modified phenol-sulfuric acid method, the yield of APCT was determined to be 3.8% (w/w) of the fresh weight, and its polysaccharide content was 98.3% (w/w) [29].

Ion-exchange and gel filtration column chromatography methods were used for the further fractionation of APCT. The APCT sample (1000 mg) was dissolved, filtered, and loaded to a column (2.4 cm × 50 cm) of DEAE-cellulose-52. The column was gradiently eluted with NaCl aqueous solution (0 M to 0.125 M), followed by 0.30 M NaOH. This process was monitored by the modified phenol-sulfuric acid method [29]. Consequently, three polysaccharide fractions (Fr1 to Fr3) were obtained. Fr2 (580 mg) eluted out of ion-exchange column with 0.125 M NaCl aqueous solution was further purified by gel filtration chromatography on a column (1.6 cm × 60 cm) of Sephadex G-100 using ultrapure water. The resulting pure polysaccharide fraction named PCT-1 was used in the subsequent chemical analyses.

### 2.4. Chemical Analysis of PCT-1

Ultraviolet (UV) spectroscopy of the PCT-1 was performed within 200 nm to 400 nm using a Lambda UV spectrometer (PerkinElmer, USA). The infrared (IR) spectra of PCT-1 was recorded within 4000 cm^−1^ to 400 cm^−1^ using a Varian 1000 Fourier-transform IR (Varian, USA) instrument with KBr pellets.

PCT-1 (20 mg) was hydrolyzed with 5 mL of 3 M sulfuric acid at 110°C for 6 h in a sealed tube. Excess sulfuric acid was neutralized with barium carbonate and the mixture was centrifuged. After the supernatant was filtered and dried, the dry solid residue was repeatedly redissolved in methanol and then dried for several times. The final solid residue (2 mg) was reacted with hydroxylamine hydrochloride (10 mg) and pyridine (0.5 mL) at 90°C for 30 min. Acetic anhydride (0.5 mL) was added, and the reaction was allowed to proceed at 90°C for 30 min. Finally, the derivatives of PCT-1 were analyzed using a Shimadzu gas chromatography/mass spectroscopy- (GC/MS-) QP2010 system (Shimadzu, Japan) equipped with an autosampler (AOC-20is).

For the GC analysis, an HP-5MS capillary analytical column (30 m × 0.25 mm × 0.25 *μ*m) and a flame ionization detector were used. The injector and detector temperatures were both set to 250°C. The column temperature was initially 60°C (held for 1 min), increased to 250°C at a rate of 5°C/min (held for 5 min), and then increased to 10°C/min at 280°C. Helium was used as the carrier gas at a constant flow rate of 1 mL/min. A sample (1.0 *μ*L) was injected in the splitless mode (split ratio = 20 : 1). The relative percentage of each analyte was calculated by comparing its average peak area with the total areas.

For the MS analysis, an electron ionization system with an ionization energy of 70 eV was used. The temperature of the ion source was 230°C. The MS transfer line temperature was set to 280°C. The acquisition mass range was 30 amu to 500 amu, and mass spectra were acquired in the scan mode at 3.33 scan/s. The Shimadzu ChemStation software was used to analyze the mass spectra and chromatograms. Monosaccharide composition was determined based on comparisons of their relative retention indices with data from the spectra of standard compounds. MS data were also compared with the known mass spectral library.

### 2.5. Animals

Female Kunming mice weighing 18 g to 22 g were provided by the Experimental Animal Center of the Third Military Medical University, Chongqing, China (animal license no. SCXK (YU) 20070003). The animals were kept in a standard polypropylene cage and maintained under a standard laboratory environment (room temperature = 22 ± 1°C, relative humidity = 55% ± 5%, and 12 h light/dark cycle). Standard diet and water were given *ad libitum*. The laboratory animal protocol was approved by the Zunyi Medical College Committee for Control and Supervision of Experimental Animals. All experimental animals were bred for 7 d before use.

### 2.6. Analyses of *In Vitro* Free Radical Scavenging

#### 2.6.1. Assay for DPPH Free Radical Scavenging

To assess the scavenging ability on the lipid-soluble DPPH free radical, a modified method was applied [30]. Phosphate buffer (2 mL, pH 6.86), fresh DPPH in ethanol solution (2 mL, 0.5 mM), and samples (0.5 mL) with different concentrations were mixed in a glass tube. The mixture was vigorously shaken and left to stand at room temperature for 30 min in the dark. The reduction of the DPPH radical was measured by monitoring the absorbance at 520 nm. The radical-scavenging activity was calculated as a percentage of DPPH discoloration by the following equation: scavenging ability (%) = [1 − (*A*_*s*_ − *A*_*sb*_)/*A*_*b*_] × 100, where *A*_*s*_, *A*_*sb*_, and *A*_*b*_ are the absorbances at 520 nm of the reaction mixture of the sample or antioxidants, sample blank, and blank, respectively. BHA and TU were used as positive controls, and the sample solution without DPPH was used as a sample blank. Ultrapure water was used as the blank control without samples or antioxidant. Three replicates were carried out for all tests.

#### 2.6.2. Assay for Hydroxyl Free Radical Scavenging

The scavenging activity for hydroxyl radical produced by the Fenton reaction was measured by the orthophenanthroline method [31] with some modifications. Phosphate buffer (4 mL, pH 7.4), orthophenanthroline in ethanol (1.5 mL, 5 mM), and FeSO_4_ (1 mL, 7.5 mM) were immediately mixed. Samples (1 mL) with different concentrations, ultrapure water (1.5 mL), and hydrogen peroxide (1 mL, 1% v/v) were added to the mixture. After incubating at 37°C for 60 min, the absorbance change of the reaction mixture caused by the color change of iron orthophenanthroline was measured at 510 nm. The hydroxyl radical scavenging activity was calculated by the following equation: scavenging ability (%) = [1 − (*A*_*s*_ − *A*_*sb*_)/*A*_*b*_] × 100, where *A*_*s*_, *A*_*sb*_, and *A*_*b*_ are the absorbances at 510 nm of the reaction mixture of the sample or antioxidants, sample blank, and blank, respectively. TU was used as a positive control, ultrapure water in place of samples and antioxidant was used as a damage control (the control in the hydroxyl radical generation system), and ultrapure water was used as a blank without sample, antioxidant, as well as hydrogen peroxide. All the tests were performed in triplicate.

#### 2.6.3. Assay for Superoxide Anion Free Radical Scavenging

Superoxide anion radical scavenging activity was determined by the 2,4-iodiphenyl-3,4-nitrophenyl-5-phenyltetrazolium chloride (NBT) method. An antioxidant kit was used as previously reported [14]. The superoxide anion free radicals generated by the xanthine-xanthine oxidase system reacted with NBT to form amethyst formazan. The absorbance of formazan in the reaction system measured at 550 nm was used to reflect indirectly the amount of superoxide anion free radicals. Therefore, the production of formazan was inversely related to the superoxide anion radical scavenging activity of the samples tested. The final results were expressed as the inhibition degree of formazan production. The percentage inhibition of superoxide anion radicals was calculated as (1 − *A*_*s*_/*A*_*b*_) × 100, where *A*_*s*_ and *A*_*b*_ are the absorbance of reaction mixture of the sample or antioxidants and blank at 550 nm, respectively. BHT was used as a positive control, and ultrapure water was used in place of samples or antioxidant as a blank. All tests were performed in triplicate.

#### 2.6.4. Assay for Ferrous Ion Chelating Activity

The chelating activity of the sample from* C. taii* on ferrous ion Fe^2+^ was measured as reported by Decker and Welch [32]. Samples (0.5 mL) with various concentrations were mixed with 1.8 mL of ultrapure water. FeCl_2_ (0.05 mL, 2 mM) and ferrozine (0.1 mL, 5 mM) were added to the mixture. Ferrozine reacted with the divalent iron to form stable magenta complex species that were very soluble in water. After 20 min at room temperature, the absorbance of the Fe^2+^-ferrozine complex in the reaction mixture was measured at 562 nm. EDTA was used as a positive control, and ultrapure water was used in place of samples or EDTA as a blank. The chelating activity of the polysaccharide fraction on Fe^2+^ was calculated as follows: chelating rate (%) = (*A*_*b*_ − *A*_*s*_)/*A*_*b*_ × 100, where *A*_*b*_ is the absorbance of the blank without samples or EDTA, and *A*_*s*_ is the absorbance with the sample or EDTA. 

### 2.7. *In Vivo* Antioxidant Defense Potential

#### 2.7.1. Experimental Design for an Aging Model Induced by *D*-Galactose

 Kunming mice were randomly divided into six groups of ten mice each. The groups were categorized as normal, model, positive control, and three APCT administration groups. The normal group was administered with 0.2 mL of normal saline s.c., and 0.5 mL of water p.o. daily. The model group was administered with 0.2 mL of 120 mg/kg *D*-galactose s.c., and 0.5 mL of water p.o. daily. The positive control group was administered with 0.2 mL of 120 mg/kg *D*-galactose s.c., and 0.5 mL of 100 mg/kg vitamin C p.o. daily. The three APCT administration groups were administered with 0.2 mL of 120 mg/kg* D-*galactose s.c., and 0.5 mL of 100, 200, and 400 mg/kg APCT p.o. daily, respectively. All mice were successively administered with the above concoctions for 45 d.

#### 2.7.2. Analysis of the Expression Levels of Endogenous Antioxidant Enzymes and MDA

 To determine the antioxidation mechanisms of APCT, its effects on antioxidant-related endogenous enzymes (i.e., SOD, GSH-Px, and CAT) and the lipid peroxidation metabolic product MDA were investigated. Blood samples were collected from the orbital venous plexuses of the mice under anesthesia just before sacrifice then all mice were sacrificed by a cervical dislocation. The brains, hearts, livers, and kidneys were rapidly excised and thoroughly washed to clear off blood. The tissues were immediately transferred to ice-cold saline and homogenized (10%) in cold saline (about 4°C). The blood and homogenate tissues were centrifuged at 3000 ×g and 4°C for 20 min. SOD, GSH-Px, CAT, and MDA in the supernatants were assessed using the respective detection kits as previously reported by Ji et al. [33].

#### 2.7.3. Immune Function Analysis Immune Organ Indices (Thymus and Spleen)

The mice of each group were fed with samples once a day for 45 d as aforementioned. After all mice were sacrificed, their bodies, thymuses, and spleens were weighed on the 46th day. The indices were expressed as the thymus and spleen weights relative to the body weight. Spleen (thymus) index = Spleen (thymus)/body weight (in mg/g).

**Lymphocyte Proliferation Assay**

Lymphocyte (T and B cells) proliferation was determined by the MTS colorimetric method [34]. The spleen lymphocytes of the sacrificed mice were isolated by centrifugation on a density gradient. A concentration of 2.0 × 10^6^ cells/mL was incubated in RPMI-1640 medium containing *L*-glutamine supplemented with 100 U/mL penicillin, 100 *μ*g/mL streptomycin, and 10% heat-inactivated fetal calf serum. HEPES buffer (10 mM) was also added to the media. The suspension cells (90 *μ*L/well) were plated onto a 96-well plate with triplicate wells. ConA and LPS (10 *μ*L, 50 *μ*g/mL) were added into each well to induce T and B lymphocytes, respectively. For the control, RPMI-1640 medium was added to each well instead of Con A or LPS. After incubation for 48 h at 37°C in a 5% CO_2_ incubator with greater than 95% humidity, the cells were treated with a Cell Proliferation MTS Assay Kit according to the manufacturer's instructions. Cells were treated with 10 *μ*L of working solution per well under a dim light. After culturing for 1 h at 37°C in the 5% CO_2_ incubator, the absorbance at 490 nm was measured using a microplate reader (Bio-Rad, USA).

**Test on the Phagocytosis of Peritoneal Macrophages**

Mice were intraperitoneally injected with 800 *μ*L of 5% (v/v) chicken red blood cells (CRBCs) for 30 min before sacrifice. Normal saline (2 mL) was injected into the abdominal cavity. The peritoneal macrophages were collected under aseptic conditions and incubated on a slide at 37°C for 30 min in a humidified incubator containing 5% CO_2_. After fixing with 4% paraformaldehyde solution, adherent cells were stained with Wright-Giemsa dye, rinsed with distilled water, dried at room temperature, and observed by light microscopy. The phagocytic ratio (PR) of the macrophages was calculated as PR = number of macrophages phagocytosing CRBCs/number of macrophages. The phagocytic index (PI) was calculated as PI = number of CRBCs phagocytosed by macrophages/number of macrophages [35].

**IgG Analysis**

Blood samples were collected from the orbital venous plexuses of mice before sacrifice. The samples were centrifuged, and the serum IgG level was measured by an automatic biochemistry analyzer (Hitachi, Japan).

### 2.8. Statistical Analyses

Experimental results were processed using SPSS 11.0 (SPSS Inc.). The data were analyzed by ANOVA and expressed as the mean ± standard deviation (SD) of triple determinations. Dunnett's *t*-test was used to compare the differences between the treated and control groups. Differences were regarded as significant at *P* < 0.05.

## 3. Results

### 3.1. *In Vitro* Free Radical Scavenging and Ferrous Ion Chelating Activities of the Aqueous Polysaccharide Fractions of *C. taii*

**Figure 1:**
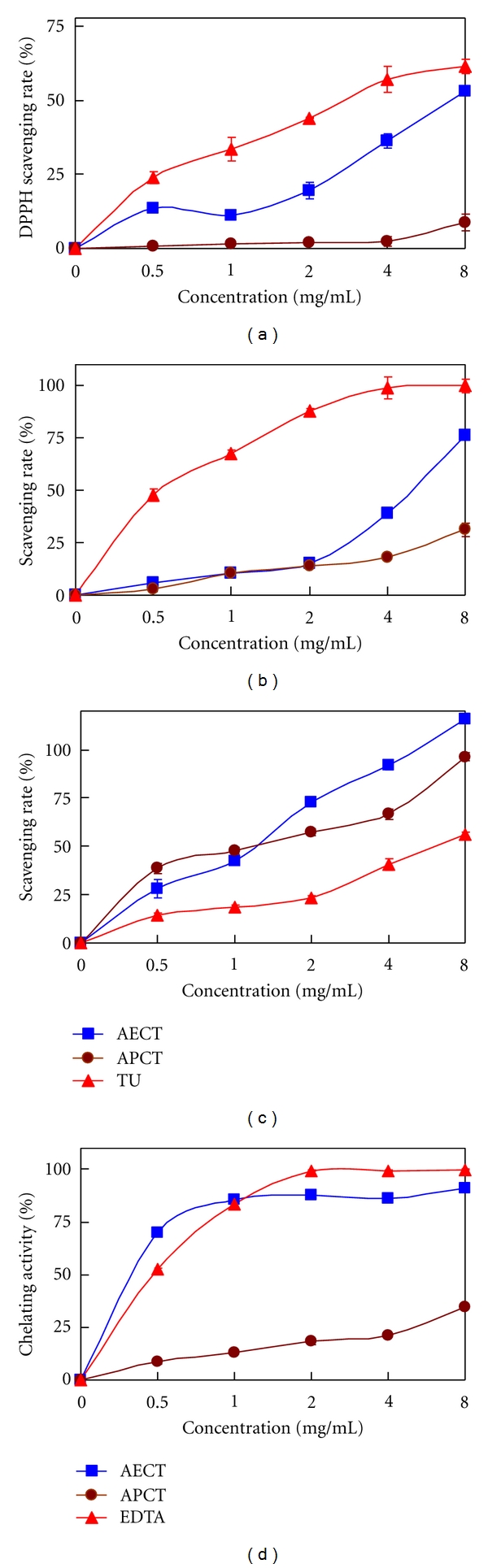
Effects of the aqueous polysaccharide fractions of *Cordyceps taii* on free radical scavenging and metal ion chelation *in vitro*. (a) DPPH free radical scavenging, (b) hydroxyl free radical scavenging, (c) superoxide anion free radical scavenging, and (d) ferrous ion chelation. AECT: aqueous extract of* C. taii*; APCT: aqueous crude polysaccharide fraction of *C. taii*; TU: thiourea; EDTA: ethylenediamine tetraacetic acid.

The antioxidant activities of the aqueous polysaccharide fractions of *C. taii*, including AECT and APCT, were evaluated based on scavenging different free radicals and chelating ferrous ion assays *in vitro*.  Figure 1 shows the antioxidant abilities at different doses. The scavenging activities of both AECT and APCT against the three different free radicals increased with increased dose from 0 mg/mL to 8.0 mg/mL in an approximately dose-dependence manner (Figures 1(a) to 1(c)). AECT and APCT, particularly at doses from 0 mg/mL to 4.0 mg/mL, exhibited relatively weak scavenging effects against both DPPH and hydroxyl free radicals compared with the positive control TU. For example, 4 mg/mL AECT and APCT showed 36.6% and 2.5%, as well as 39.2% and 18.1% scavenging effects on DPPH and hydroxyl free radicals, respectively. These values were less than the same dose of TU (57.3% and 99.1%). For these two free radicals, AECT has a more superior scavenging activity than APCT. In fact, APCT displayed a negligible effect on DPPH (Figure 1(a)). However, APCT showed more significant scavenging activities against superoxide anion free radical than TU at all doses. The scavenging effects of APCT (38.6% to 47.7%) on superoxide anion were also higher than that of AECT (27.9% to 42.4%) within 0 to 1.0 mg/mL. AECT also showed a potent scavenging activity against superoxide. For example, at 4 mg/mL, the scavenging activity of AECT was 91.9% (Figure 1(c)), which was markedly higher than the same dose of APCT (66.7%) and TU (40.7%). In terms of the 50% effective concentration (EC_50_), the scavenging potencies of AECT (EC_50_ = 2.04) and APCT (EC_50_ = 2.49) were more than 2.6-fold stronger than that of TU (EC_50_ = 6.60).  Figure 1(d) indicates that both AECT and APCT had dose-dependent chelating activities on ferrous ion, but the activity of APCT was inferior to that of AECT and only showed moderacy. The chelating activity of AECT sharply increased with increased dose from 0 mg/mL to 1.0 mg/mL, and reached a plateau of 85.4% to 91.0% at 1.0 mg/mL to 8.0 mg/mL. This result meant that AECT had a stronger chelating capacity than the positive control EDTA within 0 mg/mL to 1.0 mg/mL. EDTA only showed a slightly stronger chelating capacity (99.4% to 99.8%) at 2 mg/mL to 8 mg/mL than AECT at the same dose.

### 3.2. Antioxidant Defense Ability of APCT in *D*-Galactose-Induced Aging Mice

**Figure 2:**
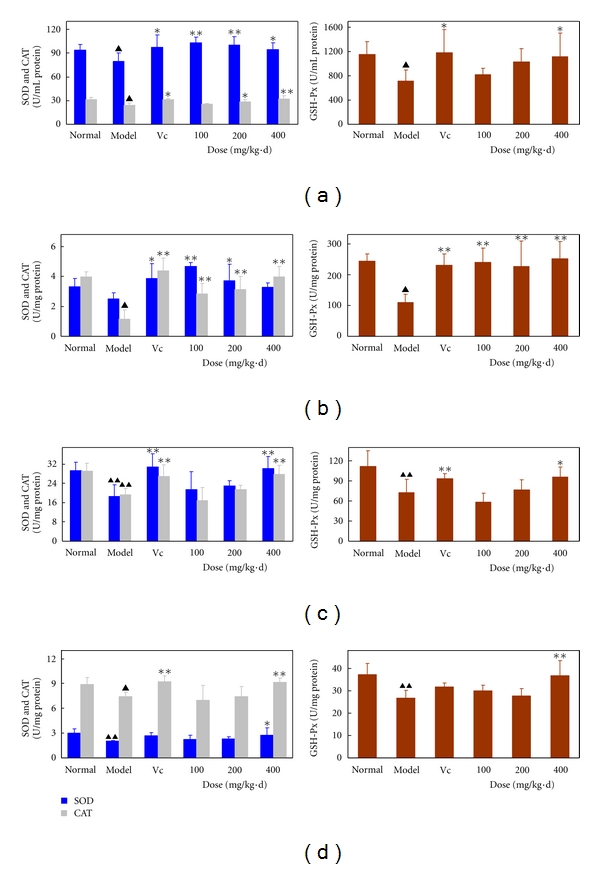
Effects of the aqueous polysaccharide fractions of *C. taii* on SOD, CAT, and GSH-Px activities of the blood (a-L/R), brain (b-L/R), liver (c-L/R), and heart (d-L/R) of *D*-galactose-induced aging mice. The normal group was administered with normal saline s.c. and water orally. The model group was administered with *D*-galactose (120 mg/kg, s.c.) and water orally. The positive control group was administered with *D*-galactose (120 mg/kg, s.c.) and vitamin C (100 mg/kg) orally. The APCT group was administered with *D*-galactose (120 mg/kg, s.c.) and APCT (100, 200, and 400 mg/kg) orally. All drugs were administered for 45 d. The values shown are the mean ± SD of 10 mice. ^▲^ *P* < 0.05, ^▲▲^ *P* < 0.01 versus normal group. *P < 0.05 and **P < 0.01 versus the model group.

**Figure 3:**
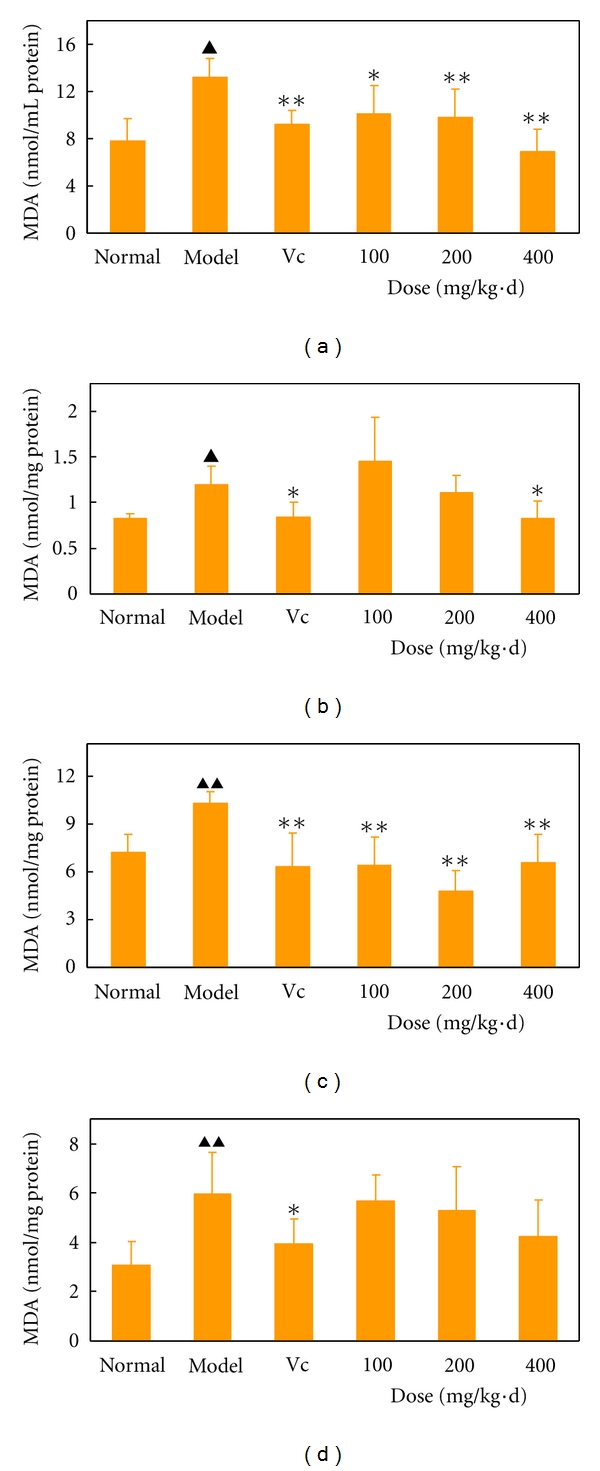
Effects of the aqueous polysaccharide fractions of *C. taii* on the MDA content in the blood (a), brain (b), liver (c), and heart (d) of *D*-galactose-induced aging mice. The normal group was administered with normal saline s.c. and water orally. The model group was administered with *D*-galactose (120 mg/kg, s.c.) and water orally. The positive control group was administered with *D*-galactose (120 mg/kg, s.c.) and vitamin C (100 mg/kg) orally. The APCT group was administered with *D*-galactose (120 mg/kg, s.c.) and APCT (100, 200, and 400 mg/kg) orally. All drugs were administered for 45 d. The values shown are the mean ± SD of 10 mice. ^▲^ *P* < 0.05 and ^▲▲^ *P* < 0.01 versus normal group. *P < 0.05, **P < 0.01 versus the model group.

Free radicals and peroxides are clearly involved in numerous physiological phenomena such as the pathogenesis of various diseases and the aging process. These biological effects of free radicals and peroxides are generally controlled *in vivo* by a wide spectrum of antioxidative defense mechanisms. In these mechanisms, the endogenous antioxidant enzymes SOD, CAT, and GSH-Px may limit the levels of reactive oxidants and the damage they inflict on the antioxidant defense system. In the present study, the effects of APCT on the activity of endogenous antioxidant enzymes (Figure 2) and the level of lipid peroxidation (Figure 3) in blood, brain, liver, and heart tissues were investigated in *D*-galactose-induced aging mice. In the model group, almost all SOD, CAT, and GSH-Px enzyme activities in the blood, brain, liver, and heart were significantly decreased compared with the normal group (*P* < 0.05  or  0.01). However, after the administration of APCT in the mice, the enzyme activities in the blood, brain, and liver almost reached higher levels than in the normal group (Figure 2). Compared with the model group, APCT at 100 mg/kg·d to 400 mg/kg·d, evidently enhanced the enzyme activities of SOD, CAT, and GSH-Px in all tissues tested, except for CAT (16.8 U/mg) and GSH-Px (58.6 U/mg) at 100 mg/kg·d of APCT in liver tissue (Figure 2(c)-L/R). The CAT (7.0 U/mg) activity in heart tissue (Figure 2(d)-L) in the administration group at 100 mg/kg·d was lower than in the model group (7.4 U/mg). The effects of APCT on the activity of these enzymes in all tested tissues were also dose dependent, except for SOD activity in blood as well as GSH-Px activities in liver and heart tissues (Figure 2). For example, the CAT activities in brain tissue steadily increased from 2.8 U/mg to 4.0 U/mg with increased APCT dose from 100 mg/kg·d to 400 mg/kg·d (Figure 2(b)-L). This increase was more than twice the enzyme activity of the model group (1.1 U/mg), but less than that of the positive control vitamin C (4.3 U/mg) at 100 mg/kg·d. Interestingly, the SOD activities in blood (Figure 2(a)-L) and brain (Figure 2(b)-L) tissues negatively correlated with the dose of APCT administration (Figure 2(a)-L). For example, the SOD activities in blood at 100, 200, and 400 mg/kg·d APCT had a descending trend (103.1, 99.9, and 94.5 U/mL in the administration group, resp.) but were markedly higher (*P* < 0.01/*P* < 0.05) than those of the model group (79.5 U/mg). The highest dose of APCT (400 mg/kg·d) significantly enhanced GSH-Px activities in all tested tissues (*P* < 0.01 or 0.05) compared with the model group (Figures 2(a), 2(b), 2(c), and 2(d)-R), and the activities were better than those in 100 mg/kg·d vitamin C. Similar results were also obtained for SOD activities in the blood and brain tissues of the administration group at 100 and 200 mg/kg·d of APCT. These activities were slightly stronger than those in 100 mg/kg·d vitamin C (Figures 2(a) and 2(b)-L).

MDA is a metabolic product of lipid peroxidation. Consequently, the lipid peroxidation level in the *D*-galactose-induced aging mice was assayed by measuring the MDA level in the blood, brain, liver, and heart tissues. The basis was the reaction of MDA with thiobarbituric acid, as shown in Figure 3. The results exhibited that the spontaneous lipid peroxidation level or MDA level in all tested tissues in the model group significantly increased compared with the normal group (*P* < 0.01  or  0.05). For the administration group, APCT within 100 mg/kg·d to 400 mg/kg·d attenuated the MDA level in all tested tissues, especially in the blood and liver tissues (Figures 3(a) to 3(d)), compared with the model group. For example, the MDA level in liver tissue in the administration group at 100, 200, and 400 mg/kg*·*d dramatically decreased to 6.4, 4.8, and 6.5 nmol/mg, respectively, which was markedly lower than the 10.3 nmol/mg in the model group (*P* < 0.01). The MDA level of the positive control vitamin C at 100 mg/kg·d was 6.3 nmol/mg (Figure 3(c)). Hence, APCT showed excellent activity against lipid peroxidation. Treatment with APCT also dose-dependently decreased the level of MDA in the blood (Figure 3(a)), brain (Figure 3(b)), and heart (Figure 3(d)) tissues of the mice with increased concentrations from 100 mg/kg·d to 400 mg/kg·d. The highest dose of APCT significantly decreased the lipid peroxidation levels in the blood (Figure 3(a)), brain (Figure 3(b)), and liver (Figure 3(c)) tissues compared with the model group (*P* < 0.01 or 0.05), in which the MDA levels were approximately or slightly higher than those in vitamin C at 100 mg/kg*·*d.

### 3.3. Effect of APCT on the Immune Function of  *D*-Galactose-Induced Aging Mice

**Figure 4:**
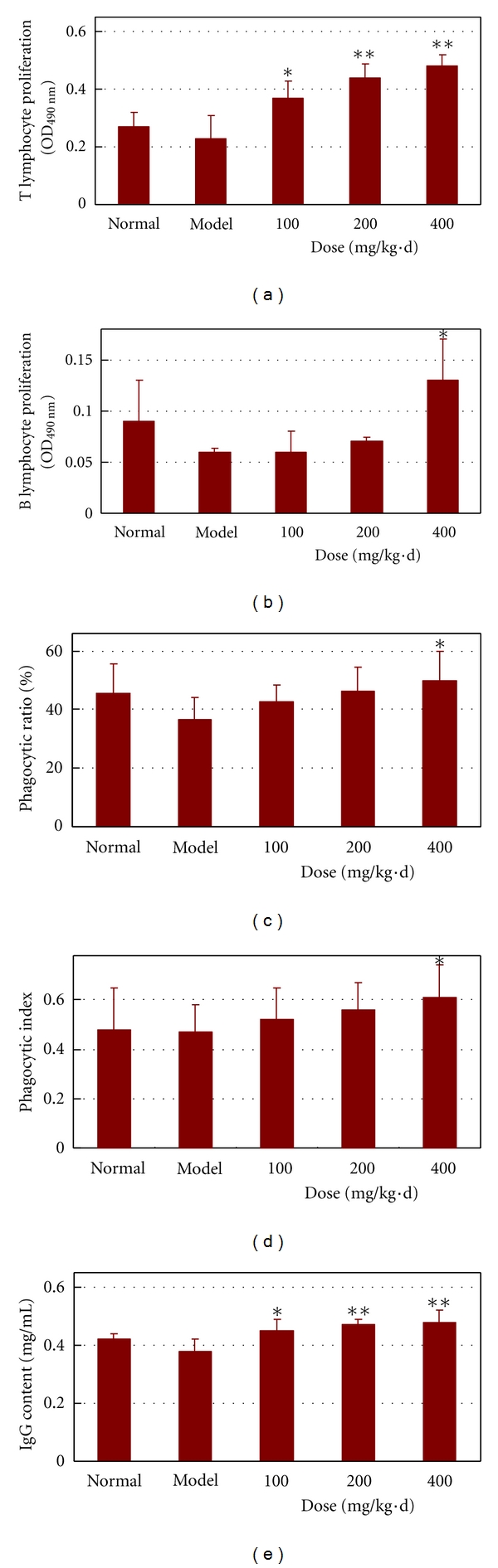
Effects of the aqueous polysaccharide fractions of *C. taii* on the immune function of *D*-galactose-induced aging mice. (a) T lymphocyte proliferation in spleen, (b) B lymphocyte proliferation in spleen, (c) phagocytic ratio of peritoneal macrophages, (d) phagocytic index of peritoneal macrophages, and (e) IgG content of venous serum. The normal group was administered with normal saline s.c. and water orally. The model group was administered with *D*-galactose (120 mg/kg, s.c.) and water orally. The positive control group was administered with *D*-galactose (120 mg/kg, s.c.) and vitamin C (100 mg/kg) orally. The APCT group was administered with *D*-galactose (120 mg/kg, s.c.) and APCT (100, 200, and 400 mg/kg) orally. All drugs were administered for 45 d. The values shown are the mean ± SD of 10 mice. *P < 0.05 and **P < 0.01 versus the model group.

Usually, organisms cannot scavenge excess free radicals by its endogenous antioxidant enzymes and chemicals, and this inability is also associated with low immunity. The thymus and spleen are the main immune organs, and immunopotentiators can increase their weights. A bigger immunity index corresponds to a stronger immune ability.  Table 1 showed that the APCT at all doses caused increased spleen and thymus indices compared with both the normal and model groups. At 200 and 400 mg/kg·d, the spleen and thymus indices of the administration group were significantly higher than those of the model group (*P* < 0.05). The results of the lymphocyte proliferation assay are shown in Figures 4(a) and 4(b). Within 100 mg/kg·d to 400 mg/kg·d, APCT dose-dependently increased the proliferation of T and B lymphocytes. In particular, there were remarkable differences between the T lymphocyte proliferations of the model and administration groups at all doses (*P* < 0.05  or  0.01, Figure 4(a)). Actually, the increased immune organs indices, especially the thymus index, suggested that APCT could enhance cell-mediated immunity through stimulating T cell formation. Additionally, at 400 mg/kg·d, the APCT also markedly promoted B lymphocyte proliferation (*P* < 0.05). Macrophages are known to play pivotal roles in nonspecific immunity due to the phagocytosis of abnormal cells or other extraneous material. The APCT dose-dependently enhanced both the PR (Figure 4(c)) and PI (Figure 4(d)) of mouse peritoneal macrophages against CRBCs at all doses. Only APCT at 400 mg/kg·d had an evidently higher phagocytosis ability than the model group (*P* < 0.05). Protective immunity comprises both cellular and humoral immunity; hence, the IgG antibody was also measured (Figure 4(e)). The APCT at all doses markedly increased the IgG content in a dose-dependent manner, and there were remarkable differences between the model and administration groups (*P* < 0.05  or  0.01).

### 3.4. Chemical Properties and Composition of APCT

**Figure 5:**
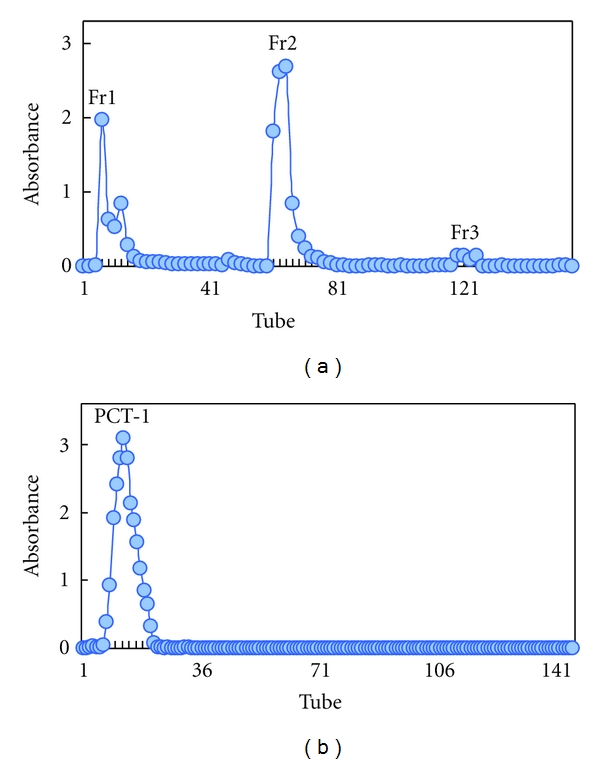
Isolation and purification of the aqueous polysaccharides of *C. taii *using chromatography columns. (a) Profile of APCT in DEAE-cellulose-52 by column chromatography (eluted with 0 M to 0.125 M NaCl and 0.3 M NaOH) and (b) profile of Fr2 by Sephadex G-100 gel column chromatography (eluted with distilled water).

**Figure 6:**
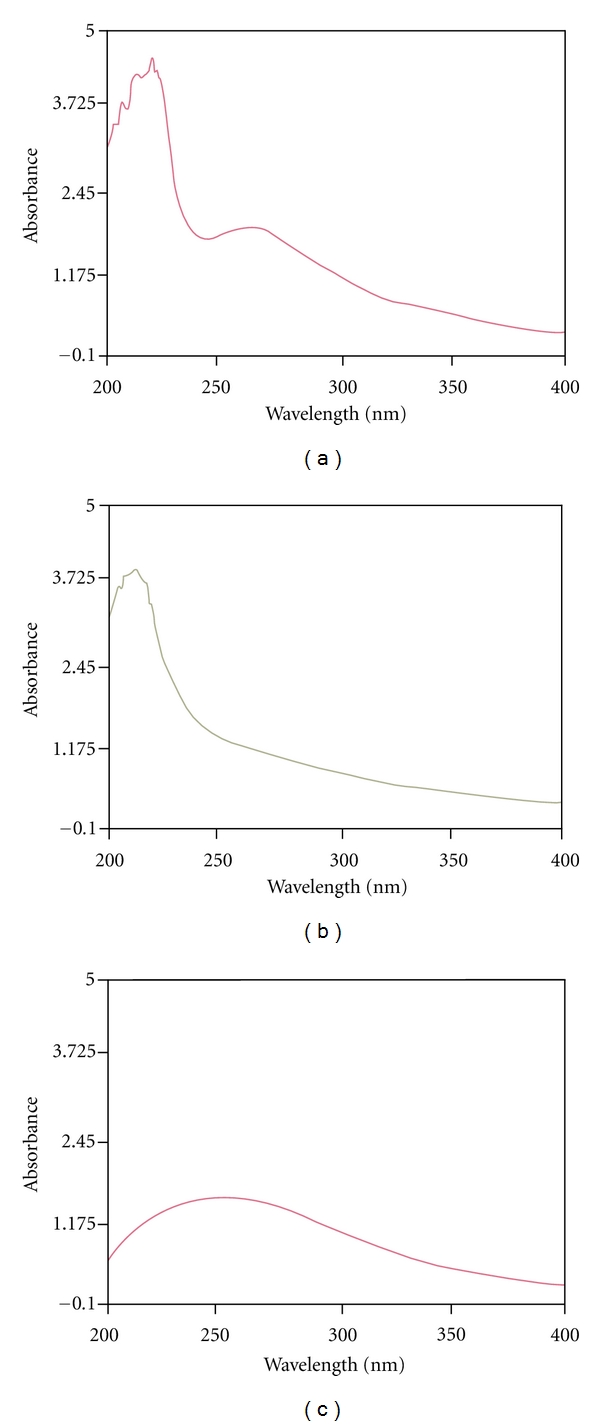
Typical UV spectra of AECT (a), APCT (b), and PCT-1 (c) isolated from *C. taii*.

APCT is a fine aqueous polysaccharide. It was obtained from *C. taii* (yield = 3.8%) by water extraction, ethanol precipitation, depigmentation, and deproteinization. After fractionation on DEAE-cellulose-52, Fr1 (240 mg), Fr2 (580 mg), and Fr3 (28 mg) were obtained from water, NaCl, and NaOH elution, respectively (Figure 5(a)). The PCT-1 fraction was obtained by gel filtration chromatography on a Sephadex G-100 column. The homogeneity of PCT-1 was elucidated by the following tests. PCT-1 (eluted with water) presented a single peak (Figure 5(b)), as detected by the phenol-sulfuric acid method, and it was homogenized under high-voltage paper electrophoresis. PCT-1, as a subfraction of AECT and APCT, showed no absorption at 280 and 260 nm by UV analysis (Figure 6(c)) compared with AECT (Figure 6(a)) and APCT (Figure 6(b)). This result implied that proteins and nucleic acids were absent in this polysaccharide fraction. 

**Figure 7:**
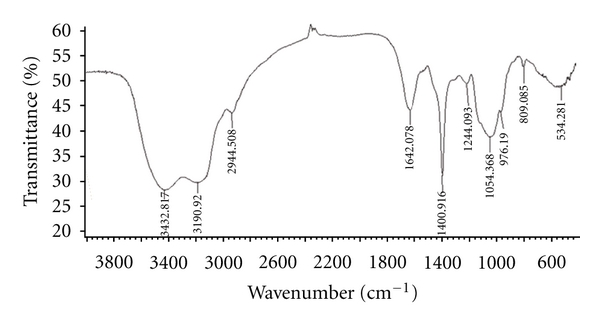
Typical IR spectrum of PCT-1 isolated from *C. taii*.

The IR absorption spectrum of PCT-1 presented main absorption bands at 3433, 3191, 2944, 1642, 1401, 1244, 1054, 976, and 809 cm^−1^ (Figure 7). The absorption bands at 840 and 891 cm^−1^ indicated that CBP-1 contained both a- and b-type glycosidic linkages in its structure. The largest absorption band at 3433 and 3191 cm^−1^ exhibited broad and intense stretching, which were assigned to hydroxyl groups. The weak stretching at 2944 cm^−1^ was attributed to the C–H bond. The bands between 950 and 1200 cm^−1^ were mostly attributed to C–O–C and C–O–H linkages. The stretching peak at 1054 cm^−1^ was suggestive of a C–O bond. The band at 1642 cm^−1^ can be attributed to water bound to the polysaccharide molecules [36]. The small absorption band at 809 cm^−1^ suggested that PCT-1 contained *α*-type glycosidic linkages in its structure [37]. Further investigation showed that PCT-1 was composed of only glucose, mannose, and galactose. Their molar ratios were 5.06 : 4.21 : 1.00 by GC/MS. 

## 4. Discussion

For the first time, the present study provided substantial evidences on the free radical scavenging and ferrous ion chelation abilities of the aqueous extract (AECT) and polysaccharide fraction (APCT) of *C. taii*. A higher sensitivity was especially exhibited against superoxide anion free radical compared with the antioxidant TU at the same dose. As aforementioned, the bioavailabilities of the most important antioxidant mechanisms are implicated in the endogenous enzymes SOD, CAT, and GSH-Px, as well as the oxidation byproduct MDA in biological systems [38]. Therefore, these recognized oxidative stress indicators were simultaneously measured in *D*-galatose-induced aging mice, and immune functions were also estimated in the present study. The findings suggested APCT exerted its robust antioxidant defense abilities through enhancing immune functions, and the activities of endogenous antioxidant enzymes, as well as inhibiting MDA production in the body.

In terms of *in vitro* drug sensitivities, there were significant differences among various free radicals. In the present study, AECT and APCT showed more significant efficacy against superoxide anion free radical than against DPPH and hydroxyl free radicals. This result was in agreement with other reports that *C. jiangxiensis* polysaccharide and *C. militaris* water extract showed weak scavenging DPPH free radical activity [14, 39]. Why? DPPH, as a stable hydrophobic free radical, may affect the scavenging abilities of hydrophilic antioxidants. A recent study has also confirmed that water extracts of both* C. militaris* and* C. sinensis *in hydrophilic systems are more effective in free radical scavenging than in hydrophobic systems [24]. For hydroxyl free radical, Halliwell and Gutteridge [40] have reported that antioxidants inhibit hydroxyl radical generation by decreasing the amount of transition metals in the Fenton reaction. In other words, there is a positive correlation between chelation reactions and the inhibition of hydroxyl free radical production. In the present study, the hydroxyl free radical scavenging (10.5% to 31.2%) and ferrous ion chelation (13.2% to 34.9%) rates of APCT at 1.0 mg/mL to 8.0 mg/mL were similarly observed. However, AECT displayed a stronger ferrous ion chelation than hydroxyl free radical scavenging activity at all tested amounts. These findings suggested that antioxidants could scavenge hydroxyl free radicals via a nonchelating reaction pathway. However, the antiradical mechanisms of AECT and APCT are still not fully understood. Yamaguchi et al. [41] have proposed that the possible antioxidant mechanism of polysaccharides may involve hydrogen donation to break chain reactions, and free radical scavenging ability results from the abstraction of anomeric hydrogen from the internal monosaccharide units of polysaccharides. Therefore, AECT and APCT are possibly good electron donors that react with free radicals to stabilize and terminate radical chain reactions by converting free radicals into more stable products.

Polysaccharides from* Cordyceps* macrofungi have evident antioxidant activities, including free radical scavenging and lipid peroxidation inhibition. Yao's group [25] has reported that the water extracts of *C. sinensis *mycelia have direct and moderate-to-potent antioxidant activities involving the scavenging of superoxide anion radical, hydroxyl radical, and DPPH radical, chelation of ferrous ion, and inhibition of linoleic acid lipid peroxidation. They also proposed that the antioxidant activities of the water extracts may be caused by a combined effect of proteins, polysaccharides, and mannitol or some other compounds in* C. sinensis* mycelia. However, Li et al. [23] have observed that the water extract of *C. sinensis* mycelia possessed a strong antioxidant activity and that partially purified polysaccharides from the water extract showed greater antioxidant activities (10-fold to 30-fold) than before purification. Therefore, polysaccharides may be regarded as the key components of the antioxidant activity of *C. sinensis* mycelia. A recent study has reported that polysaccharides of* C. militaris* mycelia showed a potent hydroxyl radical scavenging activity with an EC_50_ value of 0.638 mg/mL [17]. In the present study, AECT had a stronger free radical scavenging activity than APCT at most tested indices. Therefore, the active antiradical components of *C. taii *mycelia also contained nonpolysaccharide components. 

The antioxidant assays on APCT* in vitro* have not yet presented sufficient evidence of its antioxidant effects for treating aging-related degenerative disease closely associated with oxidative damage. In the present study, a typical mouse model whose aging was induced by *D*-galactose was used to evaluate further the antioxidant defense effect of APCT.* D*-galactose is a reducing sugar and has three metabolic pathways. Rodent chronically injected with *D*-galactose will result in disturbance of carbohydrate metabolism in the body and the formation of advanced glycation end-products *in vivo *[42]. Simultaneously, *D*-galactose also stimulates free radical generation and accumulation by a galactose-oxidase metabolic pathway [43], thus finally resulting in oxidative stress in the body. Therefore, *D*-galactose establishes the mimetic aging model on the basis of the metabolic and free radical theories of aging. Similar to natural senescence model, the *D*-galactose-induced aging mouse model shows neurological impairment, decreased activity of antioxidant enzymes, and poor immune responses [42, 44] and has been used as an animal aging model for brain aging or antiaging pharmacology research [45]. Nevertheless, the aging mechanism remains largely unknown. 

Based on the free radical theory of aging, oxidative damage is a major contributor to aging and aging-associated degenerative diseases. The most efficient defense mechanism of cells against oxidative damage mainly implicates the three endogenous enzymatic antioxidants SOD, CAT, and GSH-Px. Both SOD and GSH-Px are principally localized in the cytosolic and mitochondrial compartments of cells, whereas CAT is found in the peroxisomes of cells. Under normal physiological conditions, these enzymes can efficiently counteract oxidative damages induced by free radicals within normal cells. In contrast, there are not enough endogenous antioxidant enzymes against excessive free radicals under abnormal physiological conditions. In the present study, the changes in the production of these endogenous enzymes in different tissues were simultaneously detected in the aging mouse model. The findings revealed evident differences at the enzymatic levels of the three antioxidant enzymes in various tissues. GSH-Px presented strikingly high amounts in all tested tissues, especially in the brain, compared with SOD and CAT. Therefore, GSH-Px may exert a greater protective effect to cells, tissues, and organs against oxidative damage [46]. Usually, GSH-Px uses reduced glutathione (GSH) as the ultimate electron donor to regenerate the reduced form of selenocysteine [47], an analogue of cysteine in which selenium is substituted for sulphur. GSH is the substrate for GSH-Px, which protects cytosolic organelles from the damaging effects of the hydroperoxides formed by normal aerobic metabolism. GSH-Px, as a glutathione-depleting enzyme, is a very efficient metabolizer for reducing hydrogen peroxide and lipid hydroperoxides into harmless products at the expense of GSH, with the simultaneous conversion of GSH to oxidized glutathione (GSSG) [48], whereas GSSG can be reduced to GSH in the presence of glutathione reductase and NADPH. Therefore, any deficiency in this detoxification cycle puts the cell at risk from the potentially mutagenic effects of lipid hydroperoxides. Recently, Tsutsui et al. [49] have suggested that therapies designed to interfere with oxidative stress using GSH-Px could be beneficial to prevent myocardial remodeling and failure. In this study, APCT promoted endogenous antioxidant enzyme production or improved the enzymatic activity of endogenous antioxidant enzymes in different tissues of *D*-galactose-induced aging mice. Previous studies have found that vitamin C is an excellent antioxidant and exerts its antioxidant effect both by itself and by interacting with GSH or vitamin E [50]. Here, it was used as a positive control at a dose of 100 mg/kg·d, and indeed exerted a potent antioxidant action with a significant increase for SOD, CAT, and GSH-Px activities in blood, brain, and liver tissues of *D*-galactose-induced aging mice (*P* < 0.05  or  0.01); but some effects were less than that in the administered APCT group. More interestingly, the APCT administration groups significantly enhanced both GSH-Px and CAT activities in brain tissue (*P* < 0.01) at all tested amounts, compared with the aging model group. SOD activities in blood and brain tissues also were markedly increased after the treatment (*P* < 0.05  or  0.01). The brain SOD activity (100 mg/kg·d,) and GSH activities (100 and 400 mg/kg·d) after the treatment with APCT were higher than that in the vitamin C group. Therefore, APCT has a potential medicinal value for aging-associated brain diseases.

Lipids play crucial roles in cell metabolism by providing and storing sources of energy. However, lipid peroxidation is an important biological consequence of oxidative cellular damage [51] and can result in the formation and propagation of lipid radicals, as well as the eventual destruction of membrane lipids. Consequently, MDA, a by-product of arachidonate metabolism, is produced. Therefore, lipid protection against oxidative damage is important. MDA, a major lipid peroxidation product, is believed to be a good index of antioxidant protection [52], and it is even looked at as the marker of aging [51]. MDA resulting from tissue injury can combine with the free amino groups of proteins, producing MDA-modified protein adducts [53]. The clinical relevance of the reaction between MDA and proteins is highlighted in atherosclerosis, diabetes mellitus, and other aging-associated diseases [53]. Furthermore, MDA is also an endogenous mutagenic and carcinogenic product resulting in DNA damage and mutation [54]. It reacts with DNA to form adducts to deoxyguanosine, deoxyadenosine, and deoxycytidine. Therefore, MDA is used as a vital evaluation parameter in aging-associated diseases. In the present study, the findings indicated that MDA mainly existed in the blood and liver tissues of experimental mice and that most of the APCT administration groups very significantly inhibited MDA formation in blood and liver tissues (*P* < 0.01) compared with the aging model group. The MDA levels in the administration groups were also evidently lower than in the normal group in the liver tissues. However, vitamin C attenuated significantly the increase of MDA in all tested tissues (*P* < 0.05 or 0.01) and offered a better protection than APCT against lipid peroxidation in the body. As described above, APCT could markedly enhance the activities of different endogenous antioxidant enzymes in* D*-galactose-induced aging mice. Thus, there was a general negative correlation between the activities of endogenous antioxidant enzymes and the MDA level, suggesting that higher endogenous antioxidant enzymatic activity may protect against lipid peroxidation *in vivo*.

As aforementioned, APCT showed significant ability in scavenging superoxide anion free radical* in vitro*, enhancing endogenous antioxidant enzyme activities, and inhibiting lipid peroxidation in *D*-galactose-induced aging mice. However, the pathway by which APCT exerts its antioxidant effects *in vivo* is not yet known. At present, the free radical theory of aging proposed by Harman [1] is widely accepted, and the association of excessive free radicals with accelerated aging and a number of degenerative diseases is also well documented [55]. Furthermore, aging is accompanied by pleiotropic changes in the immune system that lead to a progressive dysregulation of cellular and humoral immune responses, also called immunosenescence [55, 56]. The cells of the immune system are particularly sensitive to changes in the oxidant-antioxidant balance because of the higher percentage of polyunsaturated fatty acids in their plasma membranes. Hence, immune cells have generally higher concentrations of antioxidant enzymes than other normal cells to defend against the attack of excessive free radicals [55]. Also, the concentration of antioxidant vitamin C in leukocytes was up to 80 times greater than that in the plasma [57], indicating that it may have functional roles in these immune system cells. Accordingly, free radicals associated with aging may be contributory factors to a depressed immune response, and that improved antioxidant levels may have an immunostimulatory action. Previous studies showed that antioxidants could not only scavenge free radicals but also improve the immune function *in vitro* and *in vivo* [10]. For example, Ohta's group has reported that vitamin C may scavenge reactive oxygen species (ROS) derived from activated neutrophils* in vitro* [50], further, it can also be a potent immune system stimulator for the proliferation, migration and chemotaxis of phagocytes, and the production of interferon, immunoglobulin, and complement [58]. Accordingly, the immune function of aging mice was systematically developed in this study. The immune system is a complex and highly regulated network of innate and adaptive immunities. Adaptive immunity involves antigen-specific receptors in B and T lymphocytes, namely, B-cell-mediated humoral and T-cell-mediated cellular immune responses [8, 10]. The results showed that APCT significantly boosted the spleen and thymus indices in the aging mice model. The increased thymus index suggested that APCT could potentiate cell-mediated immunity due to the intimate relationship between the thymus and T-cell formation, which has been documented by the spleen T-cell proliferation assay. The results showed that APCT could significantly promote spleen T-cell proliferation at all doses tested, whereas APCT could not markedly promote the spleen B-cell proliferation at all doses tested. Therefore, APCT mainly reinforced T-cell-mediated cellular immune function in aging mice. Interestingly, the critical changes characteristic of immunosenescence only occur in the T-cell population, and result in a pronounced decrease in T-cell functions, particularly T-helper cells, which affects humoral immunity and causes an impaired B-cell function [59, 60]. Thus, APCT, as a potential antioxidant and immunomodulator, could boost the T cell-mediated cellular immune responses in order to maintain homeostasis in the aging model, whereas impaired B lymphocyte response was not very sensitive to APCT, which need further research. B lymphocytes produce immunoglobulins and are activated through T-cell-dependent mechanisms or the T-cell-independent cross-linking of surface receptors by multiepitope antigens. This fact prompted us to examine IgG content in aging mice, and a positive response was observed. It is well known that aging does not affect all aspects of the immune response equally, since the influence of age on the nonspecific innate immune response mechanism is not always negative [10]. For example, Fuente's group suggested that the phagocytic cell functions did not change throughout life, while others observed a senescent decrease or increase in them [61]. Similar results were also obtained in this study, where there were no statistically significant differences between the normal and model groups on the phagocytosis of macrophage against CRBCs, and APCT administration group only showed a slight increase for the phagocytosis of macrophage at low and moderate doses compared with the model group. However, the potential mechanism responsible for the relative stable phagocytic ability of macrophage in the aging animal model still remains unclear. Anyway, it can be concluded that there is a certain causal relationship beween the antioxidant activity of APCT and its enhancing effect on immune function, although further research is needed to reveal its deep mechanism.

## 5. Conclusion

The aqueous polysaccharides of *C. taii* possess very significant scavenging superoxide anion free radical abilities and may markedly ameliorate oxidative damage in a *D*-galactose-induced aging mouse model. The amelioration is achieved by promoting the production of endogenous antioxidant enzymes and inhibiting lipid peroxidation. Hence, APCT could possibly promote the production of endogenous antioxidant enzymes by enhancing the immune system function, and concurrently, by directly scavenging excessive free radicals. The findings signify the potential of APCT as a promising source of natural antioxidants and anti-aging agents. Nevertheless, the other possible antioxidative mechanisms of action of APCT need further investigation.

## Figures and Tables

**Table 1:** Effect of APCT on the thymus and spleen indices in *D*-galactose-induced aging mice.

Group	SI	TI
Normal	4.23 ± 0.71	2.41 ± 0.74
Model	3.99 ± 0.22	2.07 ± 0.44
100 mg/kg·d	4.28 ± 1.13	2.73 ± 1.01
200 mg/kg·d	4.48 ± 0.71*	2.74 ± 0.72*
400 mg/kg·d	4.34 ± 0.34*	2.50 ± 0.59*

SI and TI represent the spleen and thymus indices, respectively. The normal group was administered with normal saline s.c. and water orally. The model group was administered with *D*-galactose (120 mg/kg, s.c.) and water orally. The positive control group was administered with *D*-galactose (120 mg/kg, s.c.) and vitamin C (100 mg/kg) orally. The APCT group was administered with *D*-galactose (120 mg/kg, s.c.) and APCT (100, 200, and 400 mg/kg) orally. All drugs were administered for 45 d. The values shown are the mean ± SD of 10 mice. *P < 0.05 and **P < 0.01 versus the model group.
